# Cephalopods as Predators: A Short Journey among Behavioral Flexibilities, Adaptions, and Feeding Habits

**DOI:** 10.3389/fphys.2017.00598

**Published:** 2017-08-17

**Authors:** Roger Villanueva, Valentina Perricone, Graziano Fiorito

**Affiliations:** ^1^Institut de Ciències del Mar, Consejo Superior de Investigaciones Científicas (CSIC) Barcelona, Spain; ^2^Association for Cephalopod Research (CephRes) Napoli, Italy; ^3^Department of Biology and Evolution of Marine Organisms, Stazione Zoologica Anton Dohrn Napoli, Italy

**Keywords:** predation, feeding behavior, prey capture

## Abstract

The diversity of cephalopod species and the differences in morphology and the habitats in which they live, illustrates the ability of this class of molluscs to adapt to all marine environments, demonstrating a wide spectrum of patterns to search, detect, select, capture, handle, and kill prey. Photo-, mechano-, and chemoreceptors provide tools for the acquisition of information about their potential preys. The use of vision to detect prey and high attack speed seem to be a predominant pattern in cephalopod species distributed in the photic zone, whereas in the deep-sea, the development of mechanoreceptor structures and the presence of long and filamentous arms are more abundant. Ambushing, luring, stalking and pursuit, speculative hunting and hunting in disguise, among others are known modes of hunting in cephalopods. Cannibalism and scavenger behavior is also known for some species and the development of current culture techniques offer evidence of their ability to feed on inert and artificial foods. Feeding requirements and prey choice change throughout development and in some species, strong ontogenetic changes in body form seem associated with changes in their diet and feeding strategies, although this is poorly understood in planktonic and larval stages. Feeding behavior is altered during senescence and particularly in brooding octopus females. Cephalopods are able to feed from a variety of food sources, from detritus to birds. Their particular requirements of lipids and copper may help to explain why marine crustaceans, rich in these components, are common prey in all cephalopod diets. The expected variation in climate change and ocean acidification and their effects on chemoreception and prey detection capacities in cephalopods are unknown and needs future research.

## Introduction

The physiology, behavior, and sensory world of cephalopods have been succesfully adapted from the luminous shallow waters to the dark and cold deep-sea, where they look for the diverse prey that meet their energy requirements. Thus, a variety of feeding behaviors have been recorded both in the wild and laboratory, in association with diverse feeding strategies (see between others, the reviews of Nixon, 1987; Hanlon and Messenger, 1996; Rodhouse and Nigmatullin, 1996). Despite being limited in number, with 845 cephalopod species described to date (Hoving et al., 2014) when compared with the very populous phylum Mollusca to which they belong, nautiluses and coleoid cephalopods (cuttlefish, squid, octopus) are an astonishing example of diversity of form and function well equipped to deal with the various marine habitats they occupy (Clarke, 1988). This is an example of how evolution can drive potential limitations in design, based to their molluscan clade, to extreme complexities (e.g., Young, 1977; Budelmann, 1995; Godfrey-Smith, 2013; Albertin et al., 2015; Allcock et al., 2015; Shigeno, 2017). Cephalopod coastal species have received more research attention because of their ease of accessibility in the field and their ability to be maintained under laboratory conditions. Most shallow water species are active visual predators with vigorous metabolic activity and sophisticated behaviors (see between others Hanlon et al., 2008; Ebisawa et al., 2011; Benoit-Bird and Gilly, 2012; Vidal et al., 2014). On the other hand, mesopelagic and deep-sea cephalopod species have been less well-studied and their feeding strategies and behaviors are not well known. Cephalopods show a significant negative relationship between metabolism and minimum habitat depth (Seibel et al., 1997; Seibel and Childress, 2000) however, in addition to buoyancy and body mass, phylogenetic position also has an influence on the metabolic rates of each individual species (Seibel and Carlini, 2001). As showed by Seibel et al. (1997), cephalopods of the family Cranchiidae as *Cranchia* and *Liocranchia* have low metabolic rates. These cephalopods live both in epipelagic waters (as subadults) and deep-sea (when adults) and do not follow the negative relationship between minimum depth and metabolic rate showed for most cephalopod species studied. The example illustrate that phylogeny is also an important factor when considering metabolic rates of individual species (Seibel and Carlini, 2001).

The following text seeks to briefly review recent advances on cephalopod predation and identify the main gaps in knowledge on this aspect of cephalopod biology and behavior. Here, we aim to briefly account for the wide spectrum of morphological, behavioral, and physiological features that cephalopods use to meet their energetic needs through predation and food intake. Along this journey we will identify possible gaps in knowledge, thus providing a short guide for future studies.

## Detecting preys

The physiology and sensory processing capabilities of cephalopods are adapted to all marine environments. Animals looking for diverse prey needed to meet energetic requirements; metabolic energetic needs that change dramatically according to the ontogenetic state, the habitat they live in and life cycle stage. A variety of feeding behaviors have been recorded in association with diverse feeding strategies (for review see Hanlon and Messenger, 1996; but see also Rodhouse and Nigmatullin, 1996), and such richness is accompanied by a sophisticated set of sensory systems (review in: Budelmann, 1994; Wells, 1994; Budelmann et al., 1997; Table 1). This developed sensory system allows them to achieve sophisticated behaviors to detect food, avoid predators and communicate between congeners in a way comparable to vertebrates. Photo-, mechano-, and chemoreceptors provide support for the collection of information about their potential prey.

**Table 1:** Biological and behavioral adaptations utilized by cephalopods for the sake of their predatory behavior.

**Predatory adaptations**		**Activities**
Senses	Eyes and vision; Epidermal hair cells; Equilibrium receptor organs for linear and angular accelerations; Epidermal tactile receptors; Contact and distance chemoreceptors; Vibration receptors and hearing	Searching for prey
Respiratory, circulatory and nervous systems	Efficient branchial ventilation; Closed circulatory system; Central nervous system; Giant fiber system	Catching prey
Physical features	Arms and tentacle; Suckers; Beaks; Jet propulsion; Skin color change	Catching and handling prey
Cognitive capabilities	Learning and memory abilities	Searching, recognition and catching prey
Hunting strategies^a^	Ambushing, Luring, Pursuit, Stalking, Speculative and Cooperative Hunting	Catching prey

*Morphological, physiological, sensory, neural and behavioral adaptations and corresponding behavioral outcomes (Activities) are listed here as deduced from several reviews (Packard, 1972; Young, 1977; Hanlon, 1988; Hanlon and Messenger, 1996; Borrelli et al., 2006; Borrelli and Fiorito, 2008)*.

a *See also Table 2*.

Probably one of the most striking features of cephalopods is their developed eye, superficially resembling that of teleost fish. It has a single nearly spherical lens with a graded refractive index, the ability to accommodate the len and a similar capacity for eye movement, showing an example of convergent evolution (Packard, 1972). The use of an adjustable pupil to control the amount of light entering the eye distinguishes the cephalopods' eye from their fish counterpart and the light-evoked pupillary constriction in cephalopods is among the fastest in the animal kingdom (Douglas et al., 2005). Among the few exceptions is the deep-sea cirrate octopod *Cirrothauma murrayi*, whose eye lacks lenses and the optic lobes are simply organized (Aldred et al., 1983), however, it is probably able to detect bioluminescence (Warrant and Locket, 2004). Most cephalopods studied have a single type of rhodopsin as a visual pigment, suggesting they are blind to color (Messenger et al., 1973; Marshall and Messenger, 1996; Mäthger et al., 2006). They can achieve spectral and color discrimination by exploiting chromatic aberration and pupil shape (Stubbs and Stubbs, 2016), but this system could work for only a narrow range of visual tasks (Gagnon et al., 2016). The giant (*Architeuthis*) and the colossal (*Mesonychoteuthis*) squids have the largest eyes in the animal kingdom, however their characteristics suggest they are mainly used for detecting and identifying bioluminescent waves generated by sperm whales during their dive into the deep, thus protecting them from potential predation, rather than detecting prey at long distances (Nilsson et al., 2012). The importance of the visual system to locate prey is also reflected in the ability for aerial capture, such as, when *Sepia officinalis* is able to attack and capture prey shown above the water surface by an experimenter (Boletzky, 1972). The complexity of the visual system of cephalopods is also achieved through extra-ocular light perception capabilities, providing an intricate network of sensory devices on their skin (see also Kingston et al., 2015; Ramirez and Oakley, 2015; Kelley and Davies, 2016). In addition, cephalopods are sensitive to polarized light and polarization vision serves to enhance the detection and recognition of prey. Squid hatchlings attack planktonic prey under polarized illumination at a 70% greater distance than under depolarized illumination (Shashar et al., 1998) and the polarization vision helps cuttlefish to see further into turbid water and to better detect prey (Cartron et al., 2013).

Sensory capabilities are not limited to vision. Cephalopods have sensory receptors that form the lateral line system, which detects gentle water currents and vibrations. Ciliated primary sensory hair cells, sensitive to local water movements, are arranged in epidermal lines located on the arms, head, anterior part of dorsal mantle and funnel (e.g., Sundermann, 1983; Budelmann and Bleckmann, 1988; Budelmann, 1994; Lenz et al., 1995) and are known to provide sensory capabilities in detecting prey (Komak et al., 2005). In fact, cuttlefish are able to catch small shrimp in the darkness and behavioral experiments showed they use the epidermal lines to detect prey (Budelmann et al., 1991).

Distant chemoreceptor organs such as, olfactory organs and rhinophores, further provide additional sensory capabilities. Olfactory organs are paired, oval shaped organs situated on either side of the head, ventrally behind the eye and near the mantle edge. Their possible role in prey detection is poorly understood. Water containing food odor (shrimp) is detected by *S. officinalis* (Boal and Golden, 1999) and embryos exposed to the odors of prey later influences prey choice in the same species (Guibé et al., 2010). Increased ventilation rates in response to prey chemicals was described for *Eledone cirrhosa* (Boyle, 1986); and positive chemotaxis for *Octopus maya* during Y-maze experiments, with amino acids (alanine, proline), nucleotids (ATP), and crab extract functioned as excitants, while betaine and taurine functioned as arrestants (Lee, 1992). The rhinophores of *Nautilus* are paired organs located below each eye and open to the exterior by a narrow pore. They are similar to the olfactory organs but are significantly larger (Basil et al., 2005).

In addition, cephalopods have contact receptors in the tentacles, sucker rims, and lips; known to allow sensing of a broad spectrum of chemical and mechanical signals. Sucker receptors are more elaborated in octopus. There are about 10,000 chemoreceptor cells in a single sucker of an octopod, but only about 100 are present in the sucker of a cuttlefish (Budelmann, 1996). The food searching habit of benthic octopods (see below Speculative pounce), that make extensive use of the arms and suckers exploring rocks and crevices, may justify this marked difference. In contrast, cuttlefish use their arms mostly for manipulating their prey (Chichery and Chichery, 1988). Contact receptors located in lips of octopus and cuttlefish are more advanced in structure and organization than those of squid. As cuttlefish and octopus are more sedentary and benthic than pelagic squid, they may rely more on tactile and chemical stimuli (Emery, 1975). Chemical receptors in cephalopods help them to locate prey and also to avoid unwanted prey. Cuttlefish were able to learn that a prey is not acceptable food, to recognize and to avoid it and, as a result, to choose a usually non-preferred prey when necessary (Darmaillacq et al., 2004).

## Ontogeny of predation: the young and the senescent

Hatchling cephalopods are of relatively large size, ranging from 0.6 (*Argonauta hians*) to 28 (*Graneledone boreopacifica*) mm mantle length (Villanueva et al., 2016), allowing the animal to start an active mode of food searching marked by the coexistence of two nutritive systems: (a) an embryonic energy in the form of yolk, and (b) a post-hatching energy provided by captured food (Boletzky, 2003). Preference for prey at hatchling when previously exposed during the latest embryonic stages (Darmaillacq et al., 2006) and visual imprinting during a short sensitive period during the first day of life (Darmaillacq et al., 2008) showed some of the available tools employed by the young cuttlefish, *S. officinalis*, to successfully capture prey and survive during the first days of life as a predator. In this species, the development of learning and predatory behavior is observed during late embryonic and early juvenile development. This occurs simultaneously with the maturation of the vertical–subvertical lobe tracts of the brain, allowing the animals to maintain a prey in the frontal field during predatory pursuit (Dickel et al., 1997). Then, during the first 3 months of life, feeding hierarchy has been reported for the same species (Mather, 1986; Warnke, 1994). A comprehensive review on this behavioral development is provided by O'Brien et al. (2016). On the other hand, in the juvenile holobenthic octopuses *O. maya*, preference to attack a prey is not obtained through previous life experience. Juvenile octopuses selected crabs as prey when individuals had previously been fed shrimp earlier in life. This could be the result of innate biological processes (Portela et al., 2014).

In squids, brain developmental differences can be found when observing the relatively large Loliginid *Sepioteuthis lessoniana* hatchlings, with a subvertical lobe of especially complicated domain structure, which may reflect an active predatory behavior (Shigeno and Yamamoto, 2005). In comparison, the minor development of higher motor centers of the small ommastrephid *Todarodes pacificus* hatchlings, suggests these animals are not active predators at this time but perhaps suspension feeders after hatching (Shigeno et al., 2001a,b). The first food and feeding strategy of the ommastrephid paralarvae before they start to actively feed on zooplankton is an unresolved question that merits further research (O'Dor et al., 1985; Vidal and Haimovici, 1998).

Diet of planktonic cephalopods in the wild is poorly understood (Passarella and Hopkins, 1991; Roura et al., 2012, 2016; Olmos-Pérez et al., 2017). Roura et al. (2016) found that *Octopus vulgaris* hatchlings targeted low abundance prey like decapod crustacean larvae independently of the zooplankton community they inhabit, thus showing a selective behavior in these patchy environments. Stable isotope ratios allowed discrimination of specific feeding strategies during ontogenesis and accumulations of metals as cadmium and mercury also reflected the ontogenetic stage in five species of cephalopods (Chouvelon et al., 2011). Externally, strong morphological changes during early life are recognized in some cephalopod groups, particularly in oegopsid squids and merobenthic octopods, associated with different habitats and feeding modes during early life. Ontogeny of prey capture develops progressively, from a simple type after hatching to an adult-like capture behavior involving structures such as, tentacles and hooks, which are absent or poorly developed in larval forms (Sweeney et al., 1992). In young ommastrephid squids, the fused tentacles forms the proboscis and its functionality, supposedly related to food capture, remain an open question that again needs future research (Uchikawa et al., 2009).

In loliginid squids, ontogeny of prey capture develops progressively, from a simple type after hatching to an adult-like capture behavior involving tentacles after 1 month of age in *Doryteuthis opalescens* raised with copepods (Chen et al., 1996). In merobenthic octopods, a positive allometric arm growth takes place during planktonic life, probably helping the animal to capture benthic prey after settlement. At the same time animals lose the oral denticles of the beaks, of which the trophic function remains unclear (Villanueva and Norman, 2008). However, observations on the external digestion and initial ingestion process in the pymy squid *Idiosepius paradoxus*, suggest that oral denticles may be used to detach the semidigested flesh from the exoskeleton of the crustacean prey (Kasugai et al., 2004). The early development of the muscular, protein-rich arm crown in merobenthic octopods is related to the decrease in lipid content of the animal, due to the relative decrease of the visceral mass, where lipids are abundant. During planktonic life, the octopus feeding behavior is that of a visual predator. The presence of prey increases the turning rate and reduces the swimming speed in *O. vulgaris* paralarvae, possibly improving the exploitation of patchy food environments in the wild (Villanueva et al., 1997).

At the other end of early life is senescence, a period coincident with the end of the single reproductive period characteristic of this group of semelparous molluscs. Chichery and Chichery (1992) found in aging *S. officinalis* signs of degeneration of the anterior basal lobe, a structure that plays an important role in the control of the predatory behavior, as indicated by previous studies by the same authors (Chichery and Chichery, 1987). In addition, they suggested that visual capacities were also affected during the aging process by reducing the attention mechanisms and also the maintenance of the predator's visual tracking behavior, concluding that the low interest in the prey shown by senescent cuttlefish may be related to the deterioration of the basal lobe and the decreasing visual input. The progressive loss of appetite in both senescent male and female octopuses is fairly well documented (see review by Anderson et al., 2002). In the brooding *O. vulgaris*, female food intake decreases about 90% and the method of predation and handling over the scarce prey changes and becomes irregular (Wodinsky, 1978). Interestingly, in the brooding female *Octopus filosus*, Wodinsky (1977) found that removal of optic glands made them cease brooding, start feeding again, and live longer than normal. This surprising behavior after removal of these glands has not been studied in other cephalopod species.

## Cephalopod feeding requirements and prey preferences

Crustaceans are present in nearly all the cephalopod diets studied to date. Teleost fish and molluscs complement their energetic needs in different proportions, depending on the species, habitat, and ontogenetic stage (see reviews of Nixon, 1987; Rodhouse and Nigmatullin, 1996). Why crustaceans seem to be an indispensable prey in the diet to sustain suitable growth for cephalopods under culture conditions, and particularly for their young stages, is a subject of current debate (Iglesias et al., 2014). Large protein and amino acid content in the diet are required to maintain positive growth, at least in shallow water cephalopod species characterized by vigorous protein metabolism and showing a relatively low quantity of lipids in their body composition. However, phospholipids, cholesterol, and long-chain polyunsaturated fatty acids (PUFA), all of them abundant in marine crustaceans, seem to play an important role. Particularly, the n-3 PUFA, due to their high demand for cell membrane synthesis where they are incorporated, due to the inability of cephalopods to synthesize them (Monroig et al., 2013; Reis et al., 2014), These findings suggest that PUFA, play an essential role in cephalopod nutrition, at least for shallow water, fast growing cephalopod species (Navarro et al., 2014). In addition, the elemental composition of natural food strongly suggests that cephalopod paralarvae and juveniles must require a food rich in copper (Villanueva and Bustamante, 2006). This fact is probably related to the haemocyanin requirements for oxygen transport, as copper is the dioxygen carrier of haemocyanin typical of crustaceans and molluscs. Again, marine crustaceans seem to play a pivotal role in the diet of cephalopods, also considering that diet of a species can change from different locations depending on the prey availability and abundance (Leite et al., 2016). The general tendency of cephalopods to prey mainly on crustaceans, fish, molluscs, and other invertebrates, such as, polychaetes, echinoderms, hydroids (Olmos-Pérez et al., 2017), and also on gelatinous fauna (Hoving and Haddock, 2017) is not followed by the deep-sea species, *Vampyroteuthis infernalis*. This species is able to fuel its low metabolism mainly on detritus (Hoving and Robison, 2012). On the other hand, as an extreme comparative example, the Giant Pacific octopus (*Enteroctopus dofleini*) and *Octopus* cf *insularis* occasionally feeds on large marine birds (Anderson and Shimek, 2014), and attacks and bite damage to the skipjack tuna (*Katsuwonus pelamis*) and yellowfin tuna (*Thunnus albacares*) inside purse seine nets have also been described for the jumbo squid *Dosidicus gigas* (Olson et al., 2006). These species are extreme examples showing the adaptive capacity of cephalopod species to obtain energy from the different marine habitats in which they live. In addition, when resources are scarce or when the density of congeners is high, cephalopods can choose cannibalism as a feeding behavior. Cannibalistic behavior has been reported from video recordings in the wild for both squids (Hoving and Robison, 2016) and octopods (Hernández-Urcera et al., 2014) independently of fishing operations, which may induce unnatural feeding behaviors. Cannibalism is common in most cephalopod species whose diet has been studied, an uncommon characteristic in the animal kingdom which may be related to their high metabolic demands. Factors influencing this unusual feeding behavior are environmental variations, population density, food availability, body size, and sexual dimorphism (Ibáñez and Keyl, 2010). In addition to visual stomach content analysis, recent tools are being used as trophic indicators and tracers in food chain pathways including stable isotope (Lorrain et al., 2011; Ohkouchi et al., 2013; Guerreiro et al., 2015), heavy metal (Bustamante et al., 1998), and fatty acid signature analysis (Pethybridge et al., 2013; Rosa et al., 2013), as well as molecular techniques (Deagle et al., 2005; Braley et al., 2010; Roura et al., 2012; Olmos-Pérez et al., 2017) and food web models (Hunsicker et al., 2010; Coll et al., 2013).

## Predatory behavioral strategies and prey capture

Until food satiation is obtained, cephalopods explore their environment looking for food. Known modes of hunting in cephalopods include ambushing, luring, stalking and pursuit, speculative hunting and hunting in disguise, among others (Table 2), described in detail by Hanlon and Messenger (1996). Behavioral observations on foraging cephalopods in their natural habitat usually come from shallow-water environments, mostly on cuttlefishes and octopuses using scuba diving. A variety of behaviors have been recorded and mimicry has been observed during octopus foraging (Forsythe and Hanlon, 1997; Hanlon et al., 2008; Krajewski et al., 2009; Caldwell et al., 2015). The sequences of foraging behavior in shallow water octopuses usually showed characteristics of a tactile saltatory searching predator, as well as a visual opportunist (Leite et al., 2009). Using acoustic techniques, coordinated school behavior during foraging was recorded at night in shallow water for jumbo squid *D. gigas*. They were observed using ascending, spiral-like swimming paths to emerge from extremely dense aggregations (Benoit-Bird and Gilly, 2012).

**Table 2:** Comparison between different hunting strategies adopted by some species of cephalopods and vertebrates (not an exhaustive list).

	**Hunting strategies**						
**CEPHALOPODS**	**Ambushing**	**Luring**	**Pursuit**	**Stalking**	**Pouncing**	**Cooperative**	**Scavenger**
*Sepia officinalis*	•	•			•		
*Euprymna scolopes*	•						
*Loligo vulgaris*	•						
*Sepioteuthis lessoniana*	•	•	•	•			
*Sepioteuthis sepioidea*	•	•	•	•	•		
*Dosidicus gigas*	•	•			•	•	
*Architeuthis dux*	•		•				•
*Mesonychoteuthis hamiltoni*	•	•					
*Vampyroteuthis infernalis*	?	•					•
*Octopus vulgaris*	•	•	•		•		
**VERTEBRATES**							
Great white shark *(Carcharodon carcharias)*	•		•	•		•	•
Nile crocodile *(Crocodylus niloticus)*	•		•	•		•	•
Eastern green mamba *(Dendroaspis angusticeps)*	•			•	•		
Golden eagle *(Aquila chrysaetos)*	•		•	•	•		•
Killer whale *(Orcinus orca)*	•		•	•		•	•
Bottlenose dolphin *(Tursiops truncatus)*	•		•			•	
Leopard *(Panthera pardus)*	•		•	•	•		•

*Hunting strategies are indicated following Curio (1976). Information included here is deduced from a series of sources including for cephalopods: Moynihan and Rodaniche (1982); Hanlon and Messenger (1996); Robison et al. (2003); Cole and Adamo (2005); Kubodera and Mori (2005); Rosa and Seibel (2010); Sugimoto and Ikeda (2013). Data from Vertebrates are presented here to attempt a possible comparison and are not exhaustive (Guggisberg, 1972; Angilletta, 1994; Martin et al., 2005; Hayward et al., 2006; Watson, 2010; Ferguson et al., 2012). “•”: hunting strategy recorded. “?”: hunting strategy probable, not recorded*.

Behavioral studies of predation in the laboratory are more detailed and abundant. The predatory strategy is part of a series of body and locomotory patterns. The visual attack is executed with great accuracy leading to a final strike, a sequence described in cuttlefishes (Messenger, 1968) and identified in different species (*Lolliguncula brevis*, Jastrebsky et al., 2017) revealing similar behavioral performances. During the attack, raised arms and dynamic skin patterns are part of these sophisticated behavioral sequences utilized presumably to deceive the potential prey and facilitate capture. Raised arms are expressed during predation when the cuttlefish has located its prey and is approaching it to reach a position suitable for attack. Arms I appear extended vertically upwards (Messenger, 1968, p. 345) and often separated in a V, each forming an S-shaped curve (review in Borrelli et al., 2006). In some cases, arms II may also be similarly raised. Raised arms are generally dark and may sway to and fro. Messenger (1968) suggests that this peculiar posture and swaying movement of the arms may act as lures, directing the prey's attention away from the tentacles. Chromatic pulses and rhythmic passing waves as been described as dynamic skin chromatic patterns of cephalopods during hunting displays. Chromatic pulses are known in cuttlefishes and also in squids and octopuses and consist of a single band of color contrast sweeping across part of the predator in a particular direction. Rhythmic passing waves are known in cuttlefishes and octopuses, involving the movement of rhythmic bands across the predator in a constant direction (see How et al., 2017 for review).

The most accurate description of full attack response of octopuses (e.g., *O. vulgaris*) is provided by Andrew Packard: “In full attack…an octopus launches itself directly toward the crab…swimming by the propulsion of water from its funnel (siphon) and without touching the bottom” (Packard, 1963, p. 39). The chromatic, postural, and locomotor components (i.e., body patterning), making up the behavior, include: (i) head and eyes raised, with the latter “wide open”; (ii) the body is or darkens to “a deep reddish-brown hue” (Packard, 1963, p. 39); (iii) the arms are outstretched or loose “with the suckers facing downwards” (Packard, 1963, p. 39); (iv) the octopus orients the siphon posteriorly, away from its target. In *O. vulgaris*, as in squid and cuttlefish, the full attack response is elicited by the initial visual recognition of an edible “object” followed by the final outcome of the attack (i.e., obtainment of food; for review see also Borrelli et al., 2006). The full attack is only one example of the variety of predatory behaviors. A full gradient of locomotor patterns appear to be exhibited. As reviewed by Borrelli et al. (2006) during crawling an octopus moves relatively slowly in contact with the ground, and may also be aided by brief swimming sequences. The animal moves along the substrate aided by the suckers of the central half of the arm, while the arms push or pull, depending on their position, to facilitate the direction of movement; this crawling may imply several arms (Finn et al., 2009) or just the posterior pair as in bipedal locomotion, also referred as walking or tiptoeing (see also Huffard et al., 2005; Borrelli et al., 2006). Crawling is adopted by octopuses to explore their surroundings and approach sites that they eventually explore for prey capture.

On the other hand, speculative hunting (or speculative pounce) is characteristic of several octopus species (see for e.g., Borrelli et al., 2006; Leite et al., 2009). While searching for prey, “the octopus moves across the bottom in a combination of swimming and crawling actions. Every 1–2 m it makes a speculative pounce, covering a rock, a clump of algae, or a small area of the bottom with its web. Pausing for a few seconds to feel under the web the octopus continues its trip” (Yarnall, 1969, p. 749).

In addition, cephalopods use different tools to enhance prey capture. For example, disguise strategies using ink during predation, has been reported recently by Sato et al. (2016) for *I. paradoxus*. These pygmy squid use ink during prey attacks in two modes: releasing ink between themselves and the prey and then attack through the ink cloud, and also releasing ink away from the prey and attacking the prey from another position. Another tool used in the darkness is the dinoflagelate bioluminescence, employed by *Euprymna scolopes* and *S. officinalis* to locate non-luminous crustaceans and fish prey (Fleisher and Case, 1995). During foraging under culture conditions, it is remarkable that cuttlefish (*Sepia pharaonis*) are able to identify the amount of prey available, discriminate prey numbers, and the following prey selection, all depending on their satiation state (Yang and Chiao, 2016). When cuttlefish detect a prey, they perform a well-known three-stage visual attack sequence of attention, positioning, and seizure (Hanlon and Messenger, 1996). Observing conspecifics during prey capture, these events do not seem to improve their predation techniques (Boal et al., 2000).

Venom is used by cuttlefishes and octopods to kill the prey and for muscle relaxation. Octopuses bored holes in the carapace, the eye or the arthrodial membrane of crustaceans (Grisley et al., 1996; Pech-Puch et al., 2016). The selection of the preferred area to inject the cephalotoxin in the crab seems to be a combination of factors related to prey and octopus size. For example, large octopuses use eye puncture less frequently than small individuals (Grisley et al., 1999). Prey handling in octopus eating bivalves showed different combinations of pulling and drilling feeding behaviors. The injection of the cephalotoxin into the bivalve and gastropod prey is associated with drilling. Drilling occurs by the combined action of radula and salivary papilla (Nixon, 1980). A combination of drilling and pulling behaviors has been reported for preying on bivalve and gastropod prey (Runham et al., 1997; Fiorito and Gherardi, 1999; Steer and Semmens, 2003; Ebisawa et al., 2011). Octopuses hold the prey within the proximal part of the arms so, they cannot use vision during most prey handling period, probably choosing the most energetic, cost effective feeding behavior based on previous experience (Anderson and Mather, 2007). In the field, other factors may influence the bivalve selection and feeding mode. McQuaid (1994) showed that mussel size selected by small octopuses (<500 g) was related to octopus weight, with small octopuses eating on small mussels because they are unable to remove large mussels attached with the byssus threads from the rocks. In addition to pulling and drilling, shell crushing has been reported as a feeding behavior for the deep-sea octopod *Graneledone* preying on gastropods, a behavior that may be favored due to their relatively larger beaks in comparison with those of shallow water octopods (Voight, 2000). It is remarkable that the elevated diversity of cephalopod hunting behaviors, almost matches the strategies adopted by vertebrate predators (Table 2). Both taxa are so diverse and remote in their phylogenetic traits, but clearly there are cases of functional (and behavioral) convergence during evolution.

## Looking for food in the cold darkness

In neritic and epipelagic cephalopods, vision is probably the main sense utilized for prey detection and capture. As light intensity decreases in deep-sea environments, low temperature reduces the metabolic demands and predator-prey distance changes (Seibel et al., 2000). In this environment, the mechanoreceptor structures in the arms, tentacles, and filaments increase in number and complexity. These metabolic and morphological changes considered to be closely related with the prey selected by deep-sea cephalopods result in feeding strategies that are more diverse in the deep-sea that previously believed. The cirrate octopods are characterized by the possession of paired filamentous cirri along the arms, of diverse length according to families, which are interspersed between a single row of suckers, and are thought to have a sensory function involved in prey detection and capture. Cirrates feed mainly on small-sized organisms with low swimming speeds including amphipods and polychaetes (Collins and Villanueva, 2006). In the cirrate *Stauroteuthis syrtensis*, blue-green bioluminescence is emitted by modified suckers without adhesive function; this has been suggested to act as a light lure to attract prey and/or mates (Johnsen et al., 1999). For *S. syrtensis* of 60 g fresh weight, a daily ration of only 1–30 calanoid copepods day^−1^ has been estimated (Jacoby et al., 2009), showing the low metabolic rate of this group of deep-sea cephalopods. In a similar way, the colossal squid (*Mesonychoteuthis hamiltoni*), the world largest invertebrate, reaching 500 kg of total weight, seems to be an ambush or sit-and-float predator that uses the hooks on its arms and tentacles to capture prey and reach a projected daily energy consumption of 45 kcal day^−1^, equivalent to only 30 g of fish day^−1^ (Rosa and Seibel, 2010). The knowledge of the diet of deep-sea squids needs further research. A comprehensive review of the main prey found in stomachs of deep-sea squids has been provided by Hoving et al. (2014).

As suggested by Young et al. (1998), the great variation in squid tentacle morphologies may reflect variation in target prey and the handling of captured food. The deep-sea squid *Grimalditeuthis bonplandi* is an extreme example: its tentacles have a very thin and fragile elastic stalk, whereas the clubs bear no suckers, hooks, or photophores. It is unknown how these tentacles are used to capture and handle their prey, as they consist on cephalopods and crustaceans (Hoving et al., 2013). Very long dorsolateral arms with photophores are present in *Lycoteuthis lorigera* males (Villanueva and Sánchez, 1993) and extremely large and filamentous arms and tentacles approximately equal in thickness and length are key characters of the genus *Magnapinna*: these reach 15–20 times the mantle length of the animal, reaching to 7 m in total length (Vecchione et al., 2001; Guerra et al., 2002). *V. infernalis* uses its two thin and retractile filaments, which may be up to nine times the body length for food capture, i.e., remains of gelatinous zooplankton, discarded larvacean houses, crustacean remains, diatoms, and fecal pellets (Hoving and Robison, 2012), thus indicating that the use of luring as a mode of hunting is probably common in deep-sea cephalopods. Also the mesopelagic *Spirula spirula* feeds mainly on detritus and zooplankton (Ohkouchi et al., 2013).

## Feeding on inert prey

As mentioned above, cephalopods do not necessarily predate exclusively on live prey. Some cephalopod species are collected in large numbers from the wild using baited traps such as, *Nautilus* (Dunstan et al., 2011) and *O. vulgaris* (Guerra, 1997) showing that scavenger behavior exists in nature. Recent development of the cephalopod culture techniques (review in Iglesias et al., 2014) allowed the use of frozen prey and/or artificial food in supporting growth during part of the life cycle in a number of species including *Nautilus*, cuttlefish (*S. officinalis, S. pharaonis, Sepiella inermis, Sepiella japonica*), squid (*Loligo vulgaris, S. lessoniana*), and octopus (*Amphioctopus aegina, O. maya, Octopus mimus, Octopus minor, O. vulgaris*). The first feeding period usually requires live crustacean prey, particularly for the delicate planktonic stages, although planktonic octopuses are able to detect, capture and ingest inert particles from the water surface (Marliave, 1981) or descending in the water column (Villanueva et al., 2002; Iglesias et al., 2007). A successful semi-humid squid paste-bound gelatine has been developed to feed *O. maya* benthic hatchlings from first feeding, showing that this species can live and reach normal growth with artificial food during the whole life cycle under laboratory conditions (Rosas et al., 2014). In other species, after a variable acclimation period, inert food is readily accepted by advanced juvenile, subadult, and/or adult stages of cephalopods under culture conditions (Vidal et al., 2014).

The training phase from feeding on live prey to inert food shows the behavioral adaptions and learning capacities of these animals under laboratory conditions. As an example noted by Nabhitabhata and Ikeda (2014), *S. lessoniana* aged 20 days can be fed sliced fish meat of two or three times the mantle length of the squid, that seize the food in the water column: when squid feed on live prey, the prey is seized by the tentacles, when the squid are fed dead feed, they change their prey capture behavior, using only their arms to seize the food and do not perform the positioning phase typical of the squid attack (Messenger, 1968). The same behavioral adaptions and prey capture modes are observed in *S. pharaonis* (Nabhitabhata, 2014a) and *S. inermis* (Nabhitabhata, 2014b) when changing from live to inert prey.

## Future challenges on cephalopod predation

In this review, we surfed through a number of important topics that require further research and possibly a dedicated effort. Research on cephalopod predatory strategies is needed in a variety of fields, from behavior to ecology. Studies of feeding behavior, nutrition, and feeding requirements are critical in order to develop the nascent cephalopod aquaculture of key species, particularly from early young stages. Studies on nutritional requirements are only at the beginning. The role of lipids on the early growth and survival of shallow water species seems more important than previously supposed and research is also needed in that field (Navarro et al., 2014). Hatchlings of 13% of the cephalopod species described to date has been obtained under laboratory conditions, most of them belonging to shallow water octopods (Villanueva et al., 2016). As this number increases in the future, new larval and juvenile predatory behavioral strategies will mostly likely be described. Similarly, the future study of deep-sea and oceanic cephalopod forms will provide further instances of novel, undescribed receptors, organs, behaviors, and modes of prey detection and capture in cephalopods (Hoving et al., 2014). In addition, whether our knowledge on diet richness of a given cephalopod species in the wild is affected or not by research effort remains to be explored; data we presented above may represent only a starting-point. The variability of conformation of cephalopod beaks and their functional relation with possible prey-items is another possible challenging avenue of research (Franco-Santos and Vidal, 2014; Franco-Santos et al., 2014). The use of modern techniques as genomics (Olmos-Pérez et al., 2017) and proteomics technologies (Varó et al., 2017), microbiota associated with different diets (Roura et al., 2017), or venom structure (Whitelaw et al., 2016) may further extend our knowledge on cephalopod diets. Some aspects, such as, the hormonal control over feeding in cephalopods are practically unknown (Wodinsky, 1977). Interactions with other species such as, intraguild predation (when species compete simultaneously for resources and interact as prey and predator), is another aspect that may need further attention in cephalopod science. The interaction between shallow water octopus and juvenile lobster is potentially an example of intraguild predation involving interference competition for refuge (Butler and Lear, 2009) but cannibalism (see above) may also be seen under this framework.

Interaction with other species, as well as competition for spatial and feeding resources will probably be modified with global change. A representative example is the case of the jumbo squid *D. gigas*. During the daytime, jumbo squids dive to the depth, suppressing metabolism in the oxygen minimum zone, an energy saving strategy in hours of prey limitation in shallow waters (Rosa and Seibel, 2008). The expected climate change expansion of deep-water hypoxia and the warming and acidification of surface waters will concentrate both prey and predators, with unknown effects on *D. gigas* predatory dynamics (Seibel, 2015). The expected variation in climate change and ocean acidification has been shown to induce complex changes in chemoreception and prey detection, including altered cue detection behaviors in some marine organisms. Changes in CO_2_ may have effects on predator handling time, satiation, and search time in coastal molluscs (Kroeker et al., 2014) as shown by increased activity in the pigmy squid *Idiosepius pygmaeus* (Spady et al., 2014). Nonetheless, the possible effects of elevated CO_2_ on chemoreception in cephalopods are unknown. The ability of carnivorous fish and sharks to detect chemical cues produced by their prey appears reduced and, as a consequence, the activity levels spent on food searching increases upon exposure to elevated CO_2_ (Cripps et al., 2011). This leads to a considerable reduction in the growth rates of sharks (Pistevos et al., 2015). Being carnivorous predators, similar effects may be expected in cephalopods, a research subject that merits future exploration.

## Author contributions

RV and GF are responsible for the conceptual design. RV, GF, and VP contributed to the writing of the manuscript.

### Conflict of interest statement

The authors declare that the research was conducted in the absence of any commercial or financial relationships that could be construed as a potential conflict of interest.
